# Why Don't CD8+ T Cells Reduce the Lifespan of SIV-Infected Cells In Vivo?

**DOI:** 10.1371/journal.pcbi.1002200

**Published:** 2011-09-29

**Authors:** Marjet Elemans, Nafisa-Katrin Seich al Basatena, Nichole R. Klatt, Christos Gkekas, Guido Silvestri, Becca Asquith

**Affiliations:** 1 Department of Immunology, Imperial College London, London, United Kingdom; 2 Laboratory of Molecular Microbiology, NIAID, National Institutes of Health, Bethesda, Maryland, United States of America; 3 School of Medicine, Emory University, Atlanta, Georgia, United States of America; Utrecht University, Netherlands

## Abstract

In January 2010 two groups independently published the observation that the depletion of CD8+ cells in SIV-infected macaques had no detectable impact on the lifespan of productively infected cells. This unexpected observation led the authors to suggest that CD8+ T cells control SIV viraemia via non-lytic mechanisms. However, a number of alternative plausible explanations, compatible with a lytic model of CD8+ T cell control, were proposed. This left the field with no consensus on how to interpret these experiments and no clear indication whether CD8+ T cells operated primarily via a lytic or a non-lytic mechanism. The aim of this work was to investigate why CD8+ T cells do not appear to reduce the lifespan of SIV-infected cells in vivo.

## Author Summary

Several studies have shown a role for CD8+ T cells in controlling SIV-infection. However, early last year two groups independently showed that depletion of CD8+ lymphocytes did not result in a measurable increase in the lifespan of productively infected cells, suggesting that direct cell killing may not be the major mechanism of antiviral activity by CD8+ lymphocytes. We investigated whether the lack of an effect on lifespan of infected cells indeed excludes a lytic role for CD8+ cells and whether a non-lytic effect of CD8+ cells, for instance by preventing new infections or blocking production of free virions, better explains the similar death rates of SIV-infected cells in animals with and without CD8+ lymphocytes. We found that, even though lytic models of CD8+ cell function are compatible with the absence of an effect of CD8+ cells on the lifespan of productively infected cells, the most likely mechanism of CD8-control in SIV-infection is via a non-lytic mechanism.

## Introduction

One of the most direct and convincing demonstrations of the importance of CD8+ T cells in controlling SIV infection in vivo is the observation that, on depleting CD8+ cells, SIV-1 viral load increases by 0.5–1 log. This observation has been made in both acute and chronic infection and has been replicated by a number of groups [1]–[3].

In 2010 two ground-breaking papers by Klatt et al and Wong et al, reported that following CD8+ cell depletion, although there was a robust increase in viral load, there was no increase in the lifespan of cells productively infected with SIV [4], [5]. That is, when the lifespan of productively infected cells was measured there was no detectable difference between control macaques with an intact CD8+ T cell response and CD8+-depleted macaques. This highly unexpected result forced a re-evaluation of the role of CD8+ T cells in SIV infection and the authors concluded that CD8+ T cells controlled viral load (since CD8+ cell depletion lead to an increase in viral load) but that control was primarily via non-lytic mechanisms (since CD8+ depletion did not increase the lifespan of infected cells).

However, it has been argued that the data are not incompatible with a lytic mechanism of CD8+ T cell control. It has been hypothesised that (i) ART-treatment may impair CD8+ T cell killing (ii) CD8+ T cell killing may occur just before the cell would die anyway [6] (iii) CD8+ T cell killing may occur prior to viral production [4], [7] or (iv) the measurements of lifespan may not be sufficiently accurate to detect a difference between depleted and control animals.

The aim of this project was to firstly investigate whether the lack of an effect of CD8-depletion on lifespan is compatible with CD8+ T cell control of SIV viraemia via a lytic mechanism and then to establish whether the data best supports a lytic or non-lytic mechanism of CD8+ T cell control.

## Results

The data of Klatt et al [4] was analysed. Briefly, 10 SIV_mac239_-infected rhesus macaques were divided into two groups of 5. One group (“control”) was treated with antiretroviral therapy (ART) alone, the other was first depleted of CD8+ lymphocytes by OKT8F mAb injection and then treated with ART (“depleted”). Depletion was performed in both early and late chronic infection (Methods). The death rate (1/lifespan) of short-lived productively infected cells was estimated from the rate of viral clearance following ART [8], [9]. Estimated death rates and model fits are shown in the supplementary information (see Table S1 and Figure S1 in Text S1).

The analysis was divided into two parts. First, we investigated whether the similar death rates of productively infected cells in control and depleted macaques rule out the possibility that CD8+ T cells act predominantly via lysis. Second, we investigated how well lytic and non-lytic mechanisms explain the longitudinal dynamics of viral load and CD4 T cell count.

### 1. Do the data rule out a lytic mechanism of action?

There are a number of explanations that potentially reconcile the lack of an effect of CD8-depletion on infected cell lifespan with a major cytolytic role for CD8+ T cells. We investigated each of these possibilities in turn.

#### (i) Impairment of CTL function by ART

**Figure 1:**
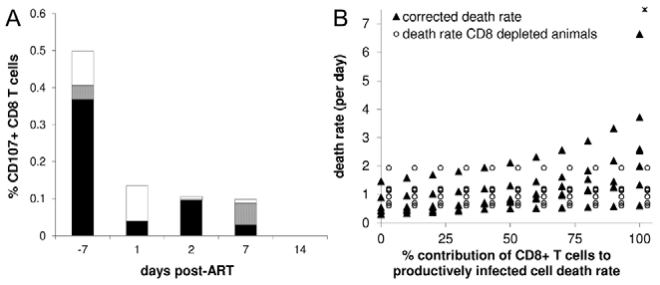
Effect of ART-treatment on CD8+ T cell function. A) Frequency of CD107+ CD8 T cells. Cells were stimulated ex vivo with either gag (black), pol (grey) or env (white) peptides and CD107-expression was measured. B) Death rate of short-lived productively infected cells corrected for reduced CTL function after ART treatment at different contribution of CD8+ T cells to total death rate (filled triangles; mean from left to right: 0.67, 0.72, 0.77, 0.83, 0.94, 1.04, 1.16, 1.32, 1.51, 1.67, 2.77d^−1^) compared to the death rate of short-lived productively infected cells in CD8-depleted animals (open dots; offset to the right, these estimates are independent of f, mean 1.08d^−1^).

It has been observed that protease inhibitors impair CD8+ T cell function [10], [11]. Whilst this phenomenon has not been reported for the reverse transcriptase inhibitors used in this study we decided to assess the impact of ART on CD8+ T cell function as this could explain the absence of an effect of CD8+ cell depletion on infected cell lifespan. The frequency of functional SIV-specific CD8+ cells was quantified by staining for IFNγ, TNFα, Il-2 and CD107 following ex vivo stimulation by SIV peptides. The frequency of IL-2+ and CD107+ cells was reduced following ART (Figure 1) consistent with [12]. Therefore, one explanation for the similar lifespan of infected cells in depleted and control animals is that ART impaired CD8+ T cell lytic function rendering control animals similar to CD8-depleted animals. To test this hypothesis we included the frequency of lytic CD8+ cells (quantified by CD107 expression) in the model and calculated the death rate of infected cells corrected for reduced CD8+ T cell function i.e. we estimated what the death rate of short-lived infected cells would have been in control animals if CD8+ T cell function was not impaired. Although this correction did increase the death rate of infected cells in control animals and thus increased the difference between control and depleted animals we only found a significant difference between the groups when we assumed all infected cell death was due to CTL killing (Figure 1). The current estimate of the fraction of total death rate attributable to CTL-killing is 20–30% (section iv and [13],[14]). We conclude that the negative effect of ART on CD8+ T cell function is unlikely to explain similar infected cell death rates in control and depleted macaques.

#### (ii) CD8+ T cell killing may occur just before the cell would die anyway or (iii) prior to viral production

Two groups have suggested that CD8+ T cells may control viral load via lytic mechanisms but have little impact on infected cell lifespan due to the infected cell lifecycle. Klenerman et al [6] suggested that if viral production increased with the duration of cell infection and the virus is highly cytopathic then CD8+ T cells may have little impact on the lifespan of the infected cell (as they kill infected cells shortly before they would die anyhow) but may have a large impact on viral load (as reducing the infected cell lifespan even by only a small amount prevents a large proportion of viral production). Althaus et al [7] proposed the opposite model: that CD8+ T cells kill infected cells prior to viral production. They suggested that, prior to de novo production of viral proteins, infected cells are a target for CD8+ T cells recognising viral proteins in the infecting particles but that later in the viral life cycle, when the cell is productively infected, they evade CD8+ T cell surveillance due to Nef-induced down-regulation of MHC class I A and B molecules. CD8+ T cells thus kill infected cells but do not shorten the lifespan of productively infected cells which is what is quantified during ART treatment. Both these possibilities are qualitatively consistent with the observations of Klatt et al, i.e. they could in principle reconcile a model in which CD8+ T cell exert their anti-viral control by a lytic mechanism with the lack of an impact of depletion on the lifespan of productively infected cells (see Figure S2 in Text S1 and [7]). However, it was clear that the reconciliation could only be achieved for a minority of parameter values. In part 2 we investigate whether these parameter values are consistent with SIV dynamics.

#### (iv) The measurements of infected cell lifespan are not accurate enough

One of the simplest explanations for the similarity in infected cell lifespan between control and CD8-depleted animals is that CD8+ T cells control viraemia by a purely lytic mechanism but the estimates of lifespan are not accurate enough to detect the increase in lifespan following depletion. There are three steps to testing this explanation:

1. estimate the increase in lifespan that would be expected following depletion if CD8+ T cells controlled viraemia by lysis.
2. estimate the errors on the measurements of lifespan of productively infected cells.
3. test if the experimental design is sufficient to observe the expected difference in lifespan between depleted and control animals.

If CD8+ T cells control viraemia by a purely lytic mechanism and target cell numbers are constant, then the rate of increase of viral load following CD8+ cell depletion is equal to the rate of lysis of productively infected cells by CD8+ T cells. We estimate that, in these 10 macaques, the geometric mean of the rate of increase in viral load is 0.25d^−1^ (see Table S2 in Text S1). So the rate of lysis of productively infected cells by CD8+ T cells is approximately 0.25d^−1^; this is similar to previous estimates of the rate of CD8+ T cell killing made using two independent approaches [13], [14]. Relaxing the assumption of constant target cells has little impact on the estimate of the rate of CTL killing (Text S1). Since it was necessary to start ART soon after CD8+ depletion there are only 3 viral load measurements from which we can estimate the rate of increase of viral load following CD8+ depletion. This makes estimating the rate of increase in viral load problematic. We explored possible sources of error in our estimates but found little evidence for significant inaccuracy (see Figure S3 in Text S1). Our estimates are similar to those reported by Wong et al [1] (geometric mean 0.39 d^−1^) but lower than the rates implied by Jin et al (geometric mean 0.8d^−1^). Differences could be due to differences in the viral strain and/or depletion protocol and timing. So, even if all of the increase in viraemia following depletion is attributable to loss of CD8+ T cell control and all of this control is exerted via lytic mechanisms, the predicted decrease in death rate following CD8-depletion is relatively small: about 0.3 per day, and may not be readily detectable.

Next, it is necessary to assess how accurately the lifespan (i.e. 1/death rate) of productively infected cells can be measured. We evaluated 4 widely used methods of estimating the errors on the measurements of infected cell lifespan (Methods). We found that the optimal method was bootstrapping the cases followed by trimming of extremes; the method employed in the original manuscript of Klatt et al (bootstrapping the residuals) tended to underestimate the error that is, it gave optimistically tight confidence intervals (see Figure S4 in Text S1).

**Figure 2:**
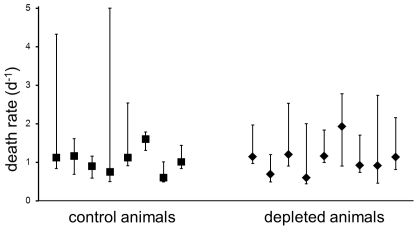
Estimated death rate of short-lived productively infected cells. Error bars represent the 95% confidence interval calculated using the optimal method (bootstrapping the cases and trimming the extremes).

**Figure 3:**
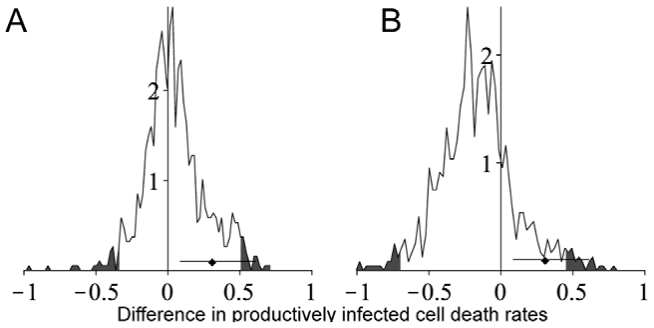
Distribution of the difference in death rate between depleted and control animals. A) early chronic infection, B) late chronic infection. These distributions represent the difference in short-lived productively infected cell death rates which are supported by the experimental data; the grey shading is the 2.5% tails. The distributions are approximately centred on zero i.e. the most likely scenario is that there is no difference in death rates of infected cells between control and depleted animals. However, the distributions are broad and large differences in the death rates, up to and including the differences which would be expected if all CD8+ T cell control was via a lytic mechanism, are also supported by the data. The filled symbol and horizontal lines represent the mean and 95% confidence interval of the difference in death rates if all CD8+ T cell mediated control was via a lytic mechanism (mean = 0.3d^−1^). A: mean difference = 0.05d^−1^ 95% CI: −0.33d^−1^ to 0.51d^−1^ B: mean difference = −0.16d^−1^ 95% CI: −0.70d^−1^ to 0.44 d^−1^.

Figure 2 shows the estimates of the death rates of short-lived productively infected cells in control and depleted macaques with errors on the estimates calculated using the optimal method. It can be seen that the errors are large and consequently, although the difference in death rates between depleted and control animals is about zero, our confidence in this difference is poor. To quantify this confidence we estimated the distribution of the difference in death rates between depleted and control animals. This yielded a broad distribution both for early chronic and late chronic infection (Figure 3). Although the distributions were centred on zero (i.e. they supported the original conclusion that there was no difference in the death rates of infected cells in the presence and absence of CD8+ T cells) they were not incompatible with a substantial difference in death rates up to and including the death rate if all viral control is attributable to CTL lysis (0.3 per day). Removing two animals with particularly large error bars did not change this result. We conclude that the estimates of productively infected cell death are not sufficiently accurate to rule out the possibility that all control of viraemia is via lytic mechanisms.

Even if the measurements of the death rates of productively infected cells in control and depleted animals are not accurate enough to detect a significant difference between the two groups intuitively we might expect to at least see a trend towards higher death rates in control animals. However, this was not the case (see Figure S5 in Text S1). This underlines the difficulty to observe differences between control and depleted animals.

Wong et al [5] also investigated whether their estimates of the death rate were sufficiently accurate to exclude a lytic role for CD8+ T cells and, using a different method, they concluded the opposite to us, i.e. that their experiments were sufficiently accurate. However, there was a minor numerical discrepancy in their calculation (Text S1), when we adjusted this we found that their conclusions were reversed and they could no longer exclude a lytic role for CD8+ T cells.

To summarize, we show that the lack of an effect of depletion on the lifespan of infected cells is not sufficient to rule out a lytic mechanism. A number of different explanations including models that assume early CTL killing in the eclipse phase; models that assume late CTL killing just before the cell would die anyhow and simple lytic models that reflect the inaccuracy of death rate measurements and thus our inability to detect differences between control and depleted animals, are all capable of explaining the lack of an effect of depletion on lifespan of infected cells. In contrast, impairment of CD8+ T cell function by ART cannot explain the similar death rates in depleted and control animals. Next we investigated how well each of the models which work in principle predict viral load and CD4+ T cell dynamics in SIV infected macaques.

### 2. Are the data more consistent with a lytic or non-lytic mechanism of action?

Having established that the estimated rates of viral clearance were not incompatible with purely lytic models of CD8+ T cell control we then investigated whether the dynamics of infection were more consistent with CD8+ T cells exerting their antiviral effects via a lytic or a non-lytic mechanism.

**Figure 4:**
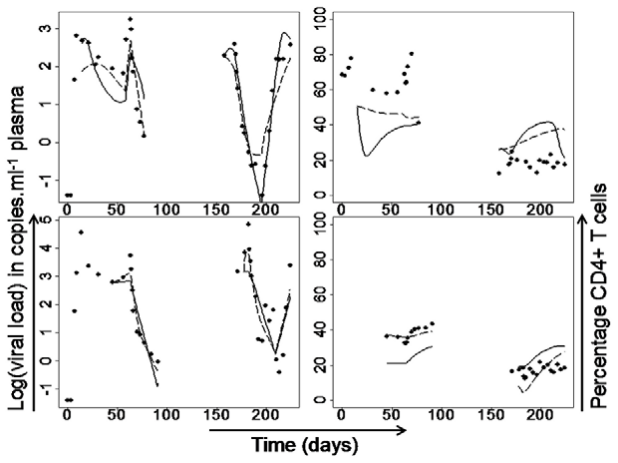
Experimental data and model fits. Experimental viral load and percentage CD4+ T cells (filled dots on left column and right column respectively) and the best fitting non-lytic model (non-lytic model ii; solid lines) and the best-fitting lytic model (lytic model i; dashed lines). Number of parameters: 10 and 8 respectively. Model selection was based in the AIC_c_, which is adjusted for differences in number of parameters. This figure shows representative data and fits, the full set can be found in Figure S6 in Text S1. Please note that for animals in Group B data for CD4+ and CD8+ T cells was only available from 45 days after infection, hence viral load data before this time point have not been fitted.

**Figure 5:**
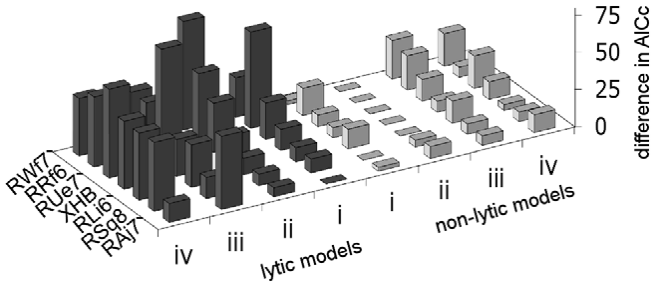
Comparison of lytic and non-lyic model fits. The AIC_c_ of the model minus the AICs of the best fitting model for that animal is shown. A large difference represents a poor fit. Overall the model in which CD8+ T cells operate via a non-lytic mechanism which reduces infection offers the best fit in 6 out of 7 cases, a lytic model only offers the best fit in 1/7 cases. The 4 lytic models are i) the basic model ii) an extension of the basic model to include two populations of productively infected cells iii) a model following Klenerman et al in which SIV is cytopathic and iv) a model following Althaus et al in which CD8+ T cell killing is limited to the early non-productive stage of the viral lifecycle. The 4 non-lytic control models were: i) a model in which non-lytic factors reduced new infection events ii) an extension of model i to include two populations of productively infected cells iii) a model in which non-lytic factors reduce virion production iv) an extension of model iii to include two populations of productively infected cells.

We considered four models of lytic control and four models of non-lytic control. The lytic control models were chosen to describe the scenarios which (in part 1 above) were found to be qualitatively consistent with the lack of an effect of depletion on infected cell lifespan. The 4 lytic control models *i*) a basic, widely used [15], [16], model of lytic control *ii*) an extension of the basic model to include two populations of productively infected cells *iii*) a model following Klenerman et al [6] in which SIV is cytopathic and *iv*) a model following Althaus et al [7] in which CD8+ T cell killing is limited to the early non-productive stage of the viral lifecycle. The 4 non-lytic control models were: *i*) a model in which non-lytic factors reduced new infection events e.g. beta-chemokines [17], [18] *ii*) an extension of model i to include two populations of productively infected cells *iii*) a model in which non-lytic factors reduce virion production [19] *iv*) an extension of model iii to include two populations of productively infected cells. To assess which of these models best describes infection dynamics we fitted each model to the time course of viral load and CD4+ T cell count in each macaque (an average of 43 time points over 224 days) and calculated the small sample Akaike's information criterion (AIC_C_) of the best fitting parameter combination (Figure 4 and 5, and Figure S6 and Table S3 in Text S1).

Although the quality of the fits is rather poor, our results show clear and consistent support for the non-lytic model in which CD8+ T cells reduce infection; none of the lytic models receive any support for most data sets. The best-fitting non-lytic model (non-lytic model ii, in which non-lytic factors reduced new infection events and there are two populations of productively infected cells) was compared with each of the lytic models in turn. In every case the non-lytic model provided a significantly better fit (lytic model i higher AICc 6/7 cases P = 0.043, mean difference in AICc = 15; lytic model ii higher AICc 6/7 cases P = 0.028, mean difference in AICc = 26; lytic model iii higher AICc 7/7 cases P = 0.018, mean difference in AICc = 40; lytic model iv higher AICc 7/7 cases P = 0.018, mean difference in AICc = 39. All P values 2 tailed paired Mann-Whitney). Furthermore the non-lytic model in which infection was reduced performed consistently better than the non-lytic model in which virion production was reduced though the differences in performance were not as large as for the comparison between lytic and non-lytic models (non-lytic model in which virion production was reduced (iv) had a higher AICc in 7/7 cases compared to the similar non-lytic model ii; P = 0.018, 2 tailed paired Mann-Whitney, mean difference in the AICc = 10).

The reason why the lytic models fail can be seen from studying the equations. In the lytic models the rate of post-ART decline in viral load is determined by infected cell death due to viral toxicity and CD8+ T cell killing. Under the CD8 depletion regime post-ART decline in viral load is solely determined by infected cell death due to viral toxicity. Thus, to predict the similar post-ART decline under the two treatment-regimes lytic models need to attribute a small role to CD8+ cells. This small role of CD8+ T cells is poorly compatible with the increase in viral load following CD8 depletion in the absence of ART. Therefore, lytic models cannot accurately fit both post-ART decline and the increase in viral load upon depletion whereas non-lytic models can. Consequently models with a non-lytic component are likely to consistently outperform similar models with a lytic component.

We conclude that although self-consistent hypotheses can be constructed in which CD8+ T cells exert their antiviral effects by lysis without a detectable impact on infected cell lifespan, these models are poorly predictive and a non-lytic model provides a better explanation of the viral load and CD4+ T cell dynamics.

## Discussion

SIV-infection of rhesus macaques is one of the best and most widely used animal models of HIV-1 infection. Indeed, the observation that CD8+ cell depletion in rhesus macaques causes an increase in viral load is frequently cited as strong evidence that CD8+ T cells control infection in HIV-1-infected humans. The finding that CD8+ T cell depletion has no discernable impact on infected cell lifespan in SIV-infected macaques is reminiscent of earlier observations in HIV-1-infected humans in which there was no relationship between infected cell lifespan and disease stage [6]. That is, in individuals with a high CD4+ T cell count and, presumably, a relatively intact CD8+ T cell response the lifespan of infected cells was very similar to individuals with a CD4 count <100. The similarity of these observations underscores the importance and generality of the work of Klatt et al and Wong et al.

We investigated a number of hypotheses to try and reconcile a major lytic role for CD8+ T cells with the observation that depletion of CD8+ T cells had little impact on the lifespan of infected cells. We found evidence that ART impairs CD8+ T cell function and allowing for this impairment increased the difference in lifespan between control and depleted animals but the difference remained small. Following Klenerman et al [6] and Althaus et al [7] we also investigated models in which CD8+ T cell killing occurred just prior to when a cell would die anyhow or prior to viral production. These models were qualitatively consistent but only for relatively narrow parameter ranges and required further assumptions (namely that SIV is highly cytopathic or that infected cells are most vulnerable to CD8+ T cell killing prior to viral production, respectively). Unexpectedly, the simplest hypothesis: that the measurements of lifespan were not accurate enough to detect the difference between control and depleted animals did offer a plausible explanation.

Depletion of CD8+ T cells results in a large increase in viral load so intuitively we expect that this implies that CD8+ T cells rapidly kill infected cells. If lysis is so rapid, it should clearly manifest as an increase in infected cell lifespan in the absence of CD8+ T cells. However, the large increase in viral load following depletion occurs over several days and, even if it is assumed to be due entirely to the removal of lytic CD8+ T cells, analysis shows that this only implies a rate of CD8+ T cell killing of 0.3 per day. That is, the rise in viraemia following CD8+ T cell depletion, indicates that CD8+ T cells are responsible for about 30% of productively infected cell death [20]. It is therefore not surprising that it is difficult to detect evidence for this relatively small contribution to cell death even in the extreme case of CD8-depleted macaques let alone in humans at different stages of infection.

Next we investigated which mechanism provides the best explanation of SIV dynamics in acute and chronic infection. This analysis found strong support for non-lytic mechanisms of CD8+ T cell-mediated control of SIV infection, in particular via blocking of infection. The models we used were fully mechanistic, low parameter models which were constrained by CD8+ T cell data and were required to fit viral load and CD4+ T cell count data simultaneously over a wide dynamic range. Consequently the divergence between observation and prediction (particularly for CD4+ T cell counts) was rather large. Clearly, none of the models used represents the true model of SIV dynamics. However, the use of the AIC enables us to select the model which contains most information of the true model, i.e. is closest to reality [21]. Furthermore, the improved performance of the non-lytic models compared to the lytic models can be understood intuitively and is likely to be a general feature of comparisons between lytic and non-lytic models.

We conclude that on their own, the lifespan estimates of Klatt and Wong cannot exclude the possibility that CD8+ T cell exert their antiviral effects via cytolysis. However, in conjunction with the failure of lytic models to compete with non-lytic models in predicting the time course of infection, the evidence favours a picture in which CD8+ T cells control infection via the production of non-lytic factors that reduce infection.

## Methods

### Ethics statement

This study was carried out in strict accordance with the Association for Assessment and Accreditation of Laboratory Animal Care guidelines and the protocol was approved by the Emory University IACUC (#062-2007Y).

### Experiment design

Ten SIV_mac239_-infected rhesus macaques were divided into two equal groups. Animals in group A were CD8+ lymphocyte depleted during early chronic phase. Animals in group B were CD8+ lymphocyte depleted during the late chronic phase. Antiretroviral therapy (ART) was given to all animals in both stages. Animals were given OKT8F (CD8-depleting mAb) for 3 consecutive days (Group A, days 58–60 after infection; Group B, days 177–179). ART (PMPA and FTC) was given for 28 consecutive days during both early and late chronic infection (starting at d63 and d168 for group A, and d63 and d182 for group B). These studies were approved by the Emory University and University of Pennsylvania Institutional Animal Care and Use Committees. Further details are provided in [4].

### Viral load

Plasma viraemia was quantified by real-time reverse-transcriptase PCR.

### SIV-specific T cell responses

SIV-specific T cell responses were measured by intracellular cytokine staining for interferon-γ, tumour necrosis factor-α, and Interleukin-2, as well as the degranulation marker CD107a, in response to pools of overlapping 15-mer peptides which spanned SIV_mac239_ gag, pol and env proteins [4].

### Estimating the death rate of productively infected cells

The death rate (1/lifespan) of productively infected cells was estimated by fitting Eq.1 to viral load after initiation of ART [4], [22].
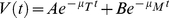
(1)where A (B) is the contribution of short-lived (long-lived) productively infected cells respectively to viral load at time zero, µ_T_ (µ_M_) is the death rate of short-lived (long-lived) productively infected cells (d^−1^) respectively.

### Basic model of HIV infection: lytic CD8+ T cells

The lytic model describes uninfected target cells (T), productively infected cells (T^*^) and free virus (V). Throughout, a dot denotes differentiation with respect to time.
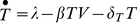
(2)

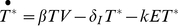
(3)

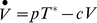
(4)Where λ is inflow of uninfected CD4+ cells (cells.d^−1^), β is infection rate, δ_T_ and δ_I_ death rate of uninfected and infected CD4+ cells respectively, k is CTL killing rate of infected CD4+ cells, p is production rate of free virions and c clearance rate of free virions (all d^−1^) The fraction of CD8^+^ T cells, E, is entered as an empirical function obtained by linear interpolation between data points. With the exception of a few points, CD8+ and CD4+ T cell measurements were available whenever viral load measurements were made.

An extension of the model with two populations of productively infected cells, T* and M*, with different death rates was also considered. This model is similar to the basic lytic model (Eq. 2 to Eq. 4) but Eq. 2 and Eq. 3 are duplicated to describe, M*, the second population of infected cells. In Eq. 4 a term representing the production of virions by this population, pM*, is added. We assume that in vivo CTL killing follows the laws of mass action. The law of mass action has been shown to hold over a wide range of effector and target cell frequencies including the cell frequencies found in our experiments [23]–[26].

### Expected decrease in infected cell death rate after CD8+ T cell depletion

We estimated the decrease in infected cell death rate consistent with the increase in viral load following CD8+ T cell depletion if CD8+ T cells operate via a purely lytic mechanism. We used the basic lytic model (Eq. 2 to Eq. 4) and, assuming constant uninfected target cells T, constant CD8+ T cell killing D prior to depletion, and a quasi-steady state between infected target cells and viral load we wrote Eq. 3 as:
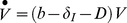
(5)Where b =  βpT/c and D = kE. Prior to depletion dV/dt = 0, so D = b-δ_I_; after depletion D = 0 and the change in viral load was described by:
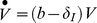
(6)If the increase in viral load following CD8-depletion was solely due to the increase in lifespan of infected cells due to prevention of CD8 killing, then the estimate of slope (b- δ_I_) gives an estimate of D. Alternative models including a model with target cell limitation and a model with an eclipse phase were also considered (Text S1).

### Comparing different methods of quantifying the error on death rate estimates

There are 4 methods that are widely used to estimate errors on parameter estimates in least-squares regression: the asymptotic covariance matrix method, bootstrapping the cases, bootstrapping the cases followed by trimming of extremes and bootstrapping the fit residuals [27]–[29]. No single method is optimal for all systems; the choice of method depends both on the model being fitted and the distribution of data. To assess the best approach for estimating the error on the death rate of short-lived productively infected cells in SIV-infected macaques we generated data in silico using the equation for viral decline after initiation of ART (Eq. 1), added random noise, fitted the model to the in silico “data” and then compared the accuracy of the 95% confidence intervals generated using the four methods. Further details are provided in Text S1.

### Distribution of difference in death rate between depleted and control animals

The distribution of bootstrap sample estimators approximates the distribution of the parameter estimator [27] so we constructed the distribution of the death rate estimates in depleted and control animals by fitting Eq. 1 to the viral load data in the 10 macaques following initiation of ART and calculated 5000 trimmed bootstrap estimates of the death rate for each animal. From these distributions we sampled, with replacement, a death rate for each animal and calculated the mean for the five animals in the control and depleted group and then the difference in the mean between the two groups. We repeated this 1000 times.

### Effect of CD8+ T cell impairment following ART treatment

To investigate if impaired CD8+ T cell function after ART-treatment, represented by reduced CD107-expression, can explain the similar death rate estimates found in CD8-depleted and control animals we explicitly included CD107-expression into the lytic model with two populations of infected cells (T* and M*). During ART treatment, infection rate β = 0, so the model reduces to:
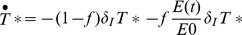
(7)

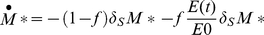
(8)

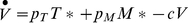
(9)Where δ_I_ and δ_S_ are the death rates of the T^*^ and M^*^ population and p_T_ and p_M_ are production rates of free virions by the T^*^ and M^*^ population respectively (all in d^−1^). Fraction f is the proportion of infected cell death attributable to CD8^+^ T cell killing; (1-f) is the proportion attributable to all other factors. Impaired CD8+ T cell function was represented by the fraction of CD107+ CD8+ T cells, E(t), relative to the pre-ART fraction of CD107+ CD8+ T cells, E0. E(t) was obtained by linear interpolation between data points. Resulting death rate estimates in control animals (i.e. corrected for CD8+ T cell impairment) were compared with death rates estimated in depleted animals using a Mann-Whitney test for different values of f.

### Alternative lytic models: assuming late virion production and late killing of infected cells

It has been suggested [6] that infected cells initially produce few virions while the bulk of virions are produced just before the cells dies. This was represented by 2 populations of SIV-infected cells: population L* consists of recently infected cells that do not produce virions and die at a negligible rate; population A* represents productively infected cells. The population of uninfected CD4+ T cells was described by Eq. 2.
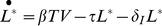
(10)

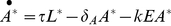
(11)

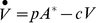
(12)Where 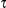 is transition rate from the L^*^ to the A^*^ population and δ_I_ and δ_A_ are the death rates of the L^*^ to the A^*^ population (all d^−1^).

### Alternative lytic models: assuming CTL killing prior to viral production

This is based on [7] and represented by two populations of SIV-infected cells: population I^*^ consists of recently infected cells which are susceptible to CD8+ T cell-mediated killing, population P^*^ consists of productively infected cells that evade CTL-killing through down-regulation of MHC-I molecules. Uninfected CD4+ T cells were described by Eq. 2.
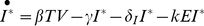
(13)

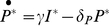
(14)

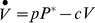
(15)Where γ is the transition rate from the I^*^ to the P^*^ population and δ_P_ is the death rate of the P^*^ population (both d^−1^).

### Non-lytic models of HIV infection

The non-lytic models share the basic description of T, T* and V dynamics (Eq. 2 to Eq. 4). However, in the non-lytic models CD8^+^ T cells do not kill infected cells (k = 0) but decrease infection rate β or virion production rate p by a fraction 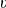:
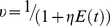
(16)


### Model fitting

The four different lytic and non-lytic models were fitted to data of virus load and total CD4^+^ T cell population, scaled to obtain equal mean. E(t) was obtained by linear interpolation of experimental data. ART-treatment was simulated by setting infection rate β = 0. To fit the models to the data we used the pseudorandom algorithm in the modFit-function of the FME-package in R [30].

### Model selection

The small sample (second-order) bias adjusted Akaike Information Criterion (AIC_c_) [21], [31] was used to compare the fit of the models. The model with the lowest AIC_c_ is considered to describe the experimental data best. As a rule of thumb we assumed considerable support for models with an AIC_c_ within two of the lowest; if models differ by three to seven AIC_c_ units from the minimum AIC_c_ there is some support for the model with the higher AIC_c_ while a difference larger than 10 suggests that the model is very unlikely [21].

## Supporting Information

Text S1 **Supporting information**
(PDF)
