# Supplementary material for: Why Don't CD8+ T Cells Reduce the Lifespan of SIV-Infected Cells In Vivo?
Source: PLoS Comput Biol. 2011 Sep 29;7(9):e1002200. doi: 10.1371/journal.pcbi.1002200 (PMC3182865; doi:10.1371/journal.pcbi.1002200)
Supplement: Text S1 — Supporting information (PDF) [file pcbi.1002200.s001.pdf]

Text S1

Estimation of the lifespan of productively infected cells

The double exponential model $vl = Ae^{-\mu_T t} + Be^{-\mu_M t}$ was fitted to log10(vl) data from day 0 to day 28 post ART. When vl was undetectable it was replaced with the limit of detection (80 copies per ml), indicated by the dashed line in Figure S1 and S2. Fits were performed by nonlinear least squares regression, confidence intervals were calculated by bootstrapping the cases and trimming the extremes. Estimates of the parameters are given in Table S1 and fits are given in Figure S1 and S2. Three animals (RPp6, RZI5 and RMm6) are missing from the late chronic calculations as they were sacrificed after the first round of ART due to severe weight loss.

id	μ_T (d-1)	μ_M (d-1)	A	B
		Gp A early chronic (CD8 depleted)		
RRf6	1.14 (0.97-1.97)	0.39 (0-0.73)	2,364,650 (1.54E+6-9.72E+6)	21,852 (78.5-5.39E+5)
RAj7	0.69 (0.49-1.20)	0.24 (0.14-0.65)	53,553,538 (1.00E+6-1E+8)	1,000,000 (2.21E+5-5.89E+7)
RLi6	1.20 (0.9-2.53)	0.22 (0-0.43)	2,072,228 (1.47E+6-9.48E+6)	31,742 (1458.7-2.50E+5)
RPp6	0.60 (0.44-2.00)	0.00 (0-0.37)	11,923,619 (5.60E+6-4.68E+7)	2,096,316 (7.93E+5-8.25E+6)
RZl5	1.16 (0.99-1.84)	0.00 (0-1.04)	2,011,364 (1.44E+6-6.57E+6)	465 (163.0-9.56E+5)
		Gp A late chronic (control)		
RRf6	1.12 (0.84-4.32)	0.00 (0-0.002)	18,334 (8.46E+3-1.17E+6)	105 (78.4-179.9)
RAj7	1.16 (0.69-1.61)	0.10 (0-0.25)	24,678,257 (7.01E+6-6.28E+7)	22,692 (1727.0-1.76E+5)
RLi6	0.90 (0.59-1.16)	0.16 (0.09-0.26)	408,343 (3.16E+5-6.88E+5)	6,733 (1297.321431.3)
		Gp B early chronic (control)		
RMm6	0.75 (0.50-5.00)	0.00 (0-0.74)	3,335,657 (0-9.14E+7)	2,337 (1-3.53E+6)
RSq8	1.12 (0.91-2.54)	0.09 (0-0.10)	222,001 (1.84E+5-3.40E+6)	5,965 (2614.0-8086.2)
RUe7	1.60 (1.31-1.79)	0.12 (0.09-0.14)	6,781,210 (5.50E+6-1.00E+7)	24,314 (16255.8-31013.7)
RWf7	0.60 (0.49-1.01)	0.12 (0.09-0.19)	1,093,995 (9.42E+5-2.20E+6)	32,170 (15191.7-1.0E+5)
XHB	1.01 (0.84-1.44)	0.09 (0.04-0.18)	522,655 (4.10E+5-1.18E+6)	1,627 (636.54296.7)
		Gp B late chronic (CD8 depleted)		
RSq8	1.93 (0.09-2.78)	0.20 (0.16-0.24)	1,850,129 (6.43E+5-3.52E+6)	28,543 (13185.7-52551.3)
RUe7	0.92 (0.74-1.70)	0.03 (0-0.22)	31,183,266 (1.00E+7-6.67E+7)	25,105 (8730.9-1.63E+6)
RWf7	0.91 (0.46-2.74)	0.12 (0.06-17)	1,446,474 (8.94E+5-8.54E+6)	53,504 (12879.7-1.64E+5)
XHB	1.13 (0.81-2.16)	0.09 (0.04-0.12)	2,053,253 (7.00E+5-9.48E+6)	971 (289.3-1820.1)

Table S1. Estimates of the death rate of the rapidly turning over subpopulation (μ_T), death rate of the slow turning over subpopulation (μ_M) and the parameters A and B. Between brackets lower and upper 95% confidence intervals are given.

Figure S1. Fits to data from Group A macaques (A) and Group B macaques (B)

A. Early chronic infection

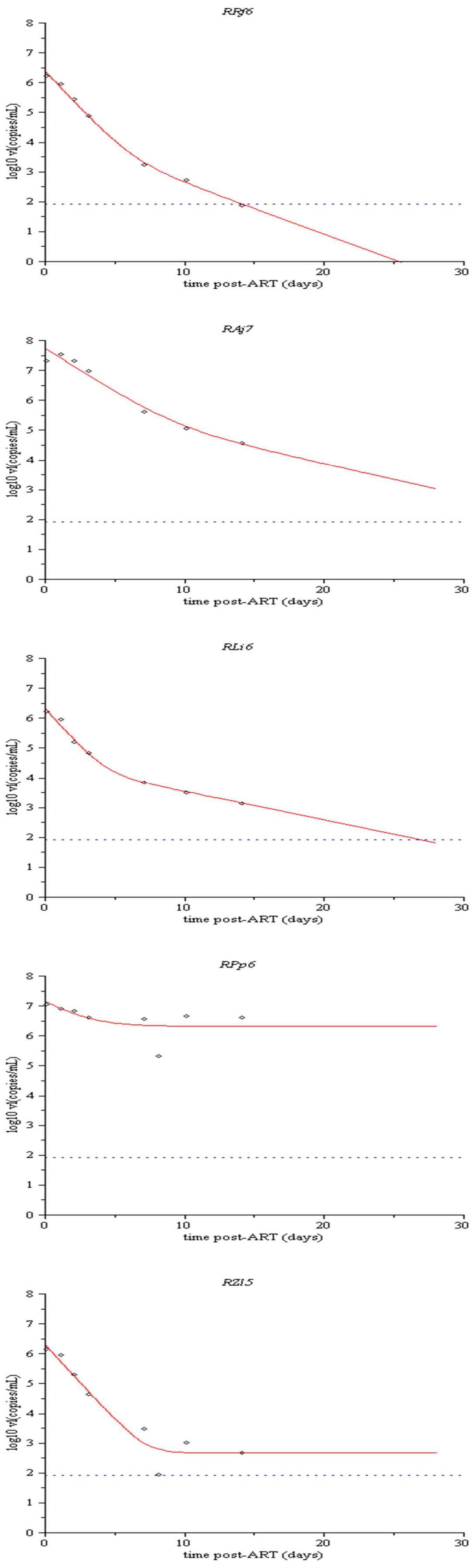

Late chronic infection

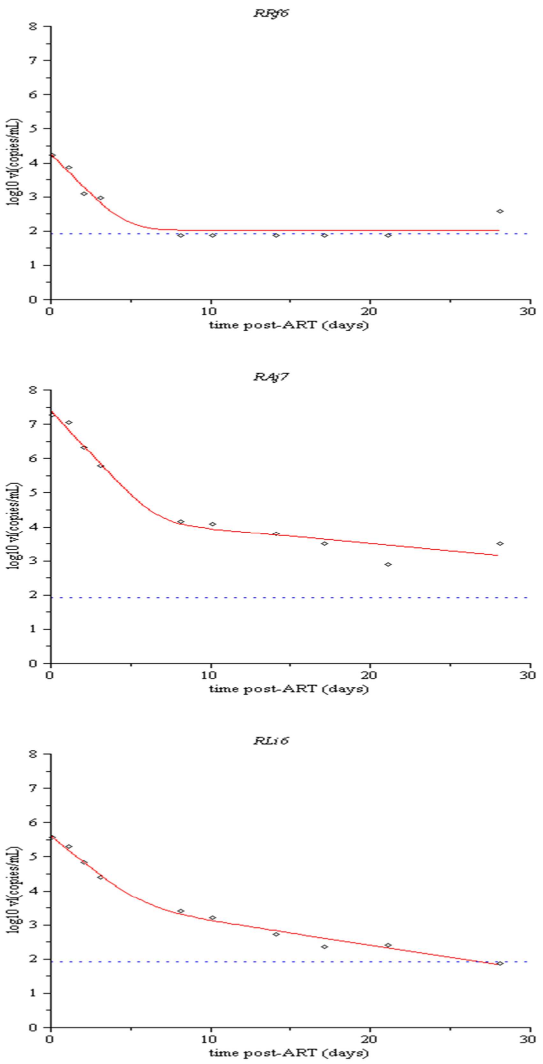

Sacrificed after first cycle of ART

Sacrificed after first cycle of ART

B. Early chronic infection

Late chronic infection

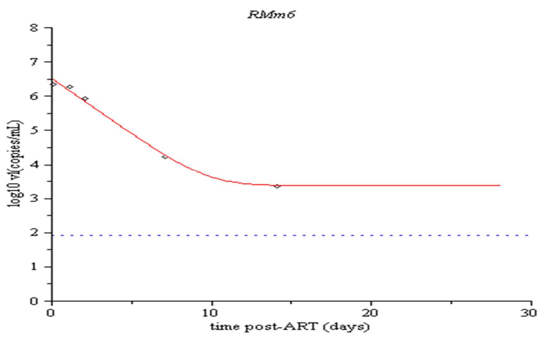

Sacrificed after first cycle of ART

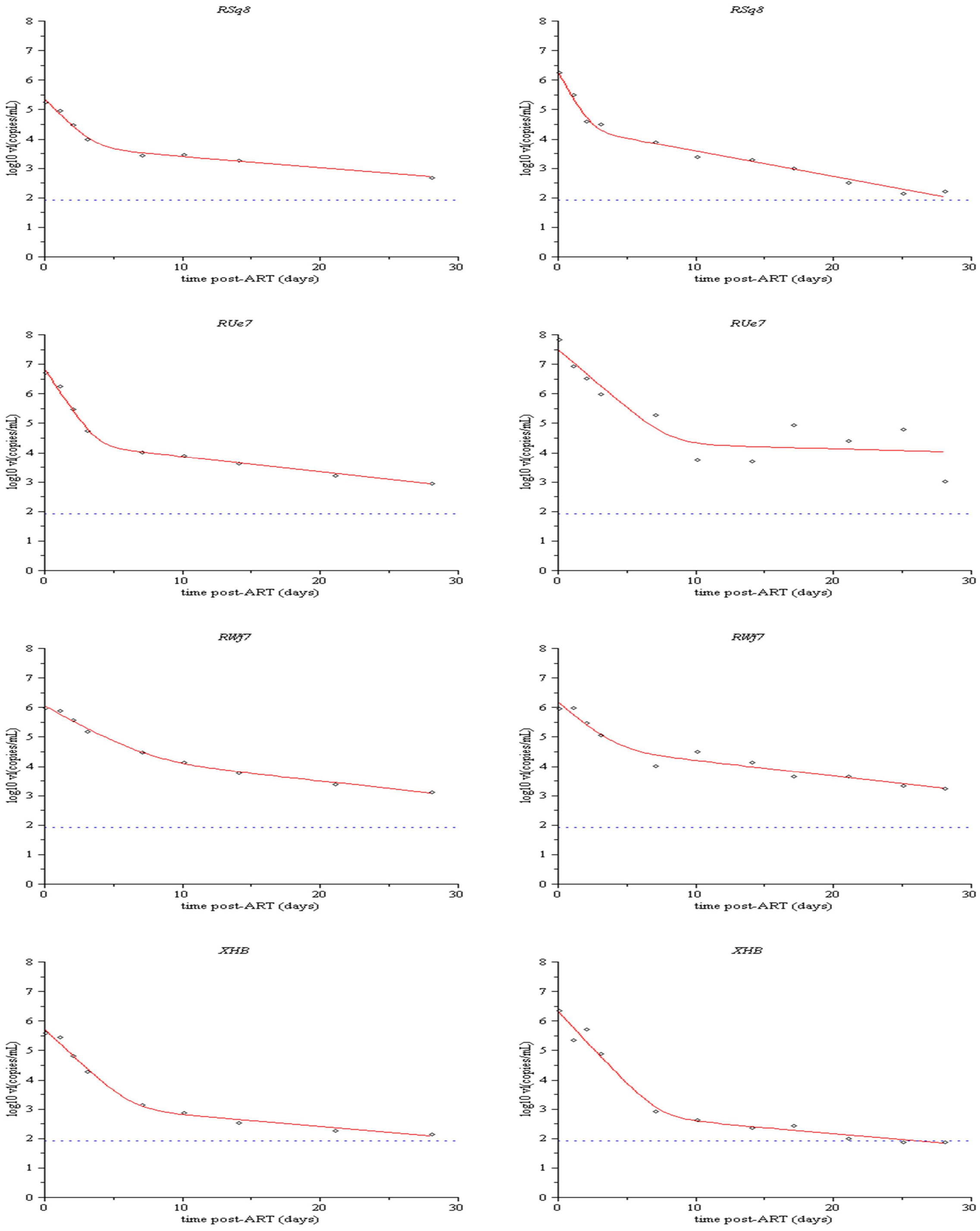

Alternative lytic model assuming late virion production and late killing

We investigated whether a model assuming late virion production and late killing of infected cells (Eqn. 10 to 12 in the main text) could reproduce basic viral load and CD4-dynamics. We defined a large set of parameter combinations by stepping at regular intervals through the parameter space for each model parameter within realistic ranges. We then selected parameter combinations which predicted steady state viral load and CD4+ T cell count between day 50 and day 58 within realistic values (change < 1.25% d^{-1} ; viral load below $7.5.10^{6}$ copies.ml^{-1}; CD4s below 1500). In addition, increase in viral load after CD8-depletion and viral decline after ART-treatment had to be within the experimentally observed range (0.08 to 0.61 d^{-1} and 0.6 to 1.93d^{-1} respectively).

Productively infected cell death was constrained to lie in the range 0.7-1.3d^{-1} and the proportion attributable to CTL killing was varied between 0 and 100%.

Less than 25% of parameters met the criteria (i.e. produced biologically realistic results for this model). For all parameter combinations that met the criteria we calculated the difference in viral decline in depleted and control animals. We found that the difference in decline after viral load between control and depleted animals is very small (less than 0.01d^{-1}, Figure S2).

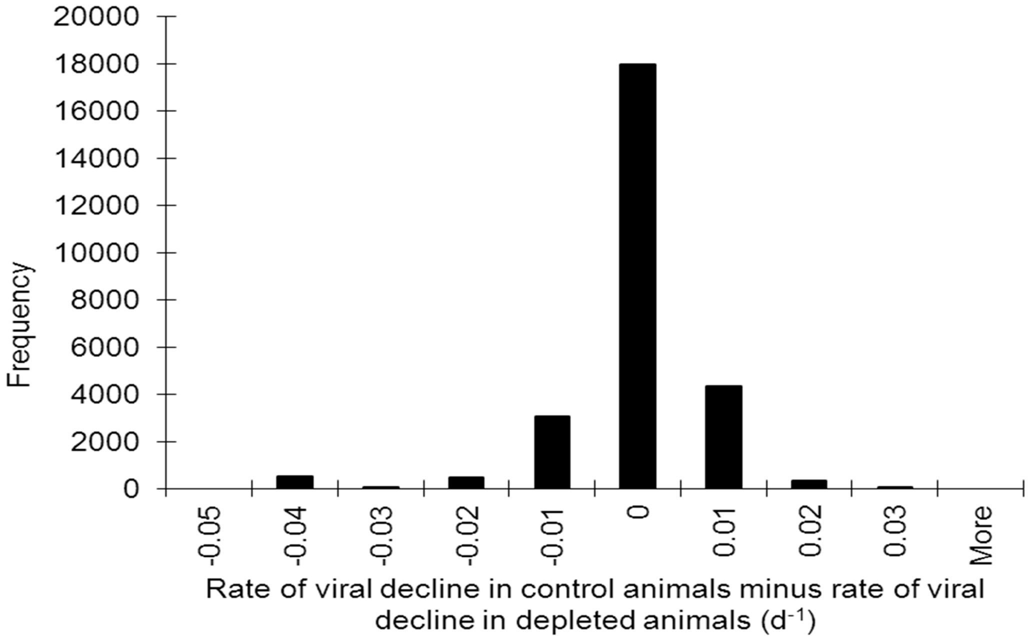

Figure S2. Frequency of the difference in viral decline after ART-treatment in depleted and control animals predicted by a lytic model assuming late virion production for a set of parameter combinations that predict steady state conditions as well as the change in viral load after either depletion or ART-treatment within the experimental range.

Rate of increase in viral load following CD8+ T cell depletion

**Animal**	**Rate of Increase in VL (d^{-1})**
RRf6	0.65
RAj7	0.15
RLi6	0.46
RPp6	0.08
RZl5	0.23
RSq8	0.28
RUe7	0.34
RWf7	0.10
XHB	0.44
**Mean**	**0.30**
**Geometric mean**	**0.25**

Table S2 Rate of increase in viral load following CD8-depletion.

Our estimates are similar to those reported by Wong et al [3] (in three fully depleted macaques they found rates of 0.32, 0.35, 0.55d^{-1}) but lower than the rates implied by Jin et al (geometric mean 0.8d^{-1}). These differences could be due to differences in the viral strain and/or depletion protocol and timing.

Since it was necessary to start ART soon after CD8+ depletion there are only 3 viral load measurements from which we can estimate the rate of increase of viral load following CD8+ depletion prior to ART (group A: day 56, 59, 63, group B: 171, 178, 179). This makes estimating the rate of increase in viral load problematic. If the rate of increase in viral load is higher than we estimated then it might be possible to reject the lytic model as we would predict a larger increase in lifespan of infected cells in depleted animals which would not be compatible with the observations. We therefore explored two possibilities which could cause us to underestimate the rate of increase in viral load:

We were concerned that if viral load reaches a new plateau before the final data point we would underestimate the rate of increase in viral load. To test this possibility we calculated the rate of increase in viral load between the first and second time point and between the second and third time point. If viral load had plateaued prior to the final time point leading to an underestimate in the rate of increase in viral load then this would be manifest as a slower rate between the last two time points than between the first two. We did not find any evidence for this. In approximately half of cases (4 out of 9) the rate of increase was greater between the last two time points than between the first two (Figure S3).

A second possible cause of an underestimate in viral load is that the first time point is not immediately prior to depletion (which occurred on day 58 in Gp A and day 177 in Gp B). We therefore also calculated the rate of increase in viral load which would result if the viral load observed at the first time point was observed the day before depletion. This had little impact on the estimate of the rate of increase of viral load increasing the geometric mean from 0.25d^{-1} to 0.31d^{-1}.

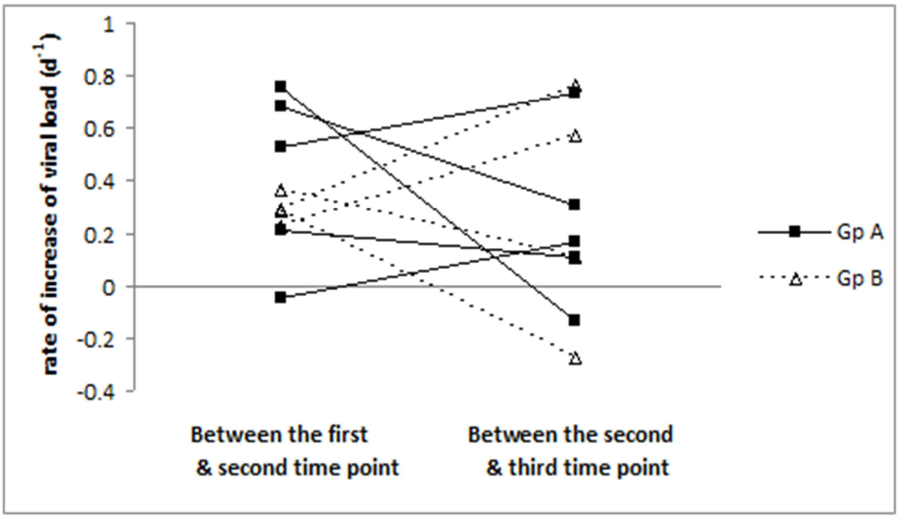

Figure S3. The rate of increase in viral load following depletion. We found no evidence to reject the null hypothesis that the rate of viral increase was the same between the first and second time point as between the second and third time point (p=1, Sign test).

Alternative models of the increase in vl following CD8+ T cell depletion

We also investigated two alternative models to quantify the rate of CD8+ T cell lysis implied by the increase in viral load following CD8+ T cell depletion: a model with target cell limitation and a model with an eclipse phase. In both cases, due to the shortage of data points in each animal post-depletion but prior to ART, all data were fitted simultaneously with one (population) estimate of each free parameter.

<u>Target cell limitation</u>

The basic lytic model (Eqn 2-4 in the main text) was used. To reduce free parameters, we assume that, following depletion no new uninfected targets are produced so the number of uninfected targets at time t is the number at time zero (i.e. at the point of depletion) minus the number of infected cells $T(t)=T(0)-T^*(t)$, furthermore we assume quasi steady state between free virions and infected cells so the system is governed by one equation:

$$\frac{dV}{dt}=\frac{p}{c}\beta T(t)V(t)-\delta_I V(t)-kE(t)V(t).$$

Prior to depletion $dV/dt=0$ so $T^*(0)=(d_I+D)c/pb$; where $D=kE(0)$. After depletion $E(t)=0$ so

$$\frac{dV}{dt}=\frac{p}{c}b\left[T^*(0)-p\frac{V(t)}{c}\right]V(t)-d_I V(t)$$

Which can be solved analytically to give

$$V(t)=\frac{V(0)e^{Dt}}{1-\Gamma V(0)[1-e^{Dt}]}$$

Where $\Gamma = {}^{\beta V(0)}\!/\!_{D}$.

Fitting this model to the data yields D=0.30d^{-1} and Γ=0 target cell^{-1}d^{-1}.

<u>Eclipse model</u>

The basic lytic model (Eqn 2-4 in the main text) was adapted to include an eclipse phase of 1 day between infection of a cell and production of free virions. So now T* represents productively infected cells and

$$\frac{dT^*(t)}{dt}=bT(t-\tau)V(t-\tau)-d_I T^*(t)-kE(t)T^*(t)$$
$$\frac{dV(t)}{dt}=pT^*(t)-cV(t)$$

Where τ is the length of the eclipse phase (τ =1 day) [1]. As before we assume a constant number of uninfected target cells $T(t) = T$, constant CD8+ T cell killing prior to depletion $D = kE(0)$ and a quasi steady state between infected cells and free virus yielding

$$V(t) = V(0)(\delta_I + D - De^{-\delta_I t}) / \delta_I \qquad t \in [0, \tau]$$

$$V(t) = V(0)(\delta_I + D - De^{-\delta_I \tau})e^{D(t-\tau)} / \delta_I \qquad t > \tau$$

We eliminated δ_I using the estimated lifespan of a productively infected cell of 1 day (i.e. $\delta_I + D = 1$) [2]. Fitting this model to the data yielded $D = 0.32d^{-1}$.

Fitting the simple lytic model (either with individual parameters as reported in the manuscript or with a population parameter to provide a direct comparison with the approach used here) gives very similar estimates of the rate of CTL killing of productively infected cells (individual parameters geometric mean: $0.25d^{-1}$, population parameter: $0.30d^{-1}$)

In summary, neither alternative model had much impact on our estimate of the rate of CTL killing implied by the increase in viral load following depletion.

Finding the optimal method to estimate errors on the lifespan measurements

We evaluated four widely used methods for calculating the confidence interval on parameter estimates: the asymptotic covariance matrix method, bootstrapping the cases, bootstrapping the cases followed by trimming of the extreme parameter estimates in the 2.5% tails and bootstrapping the residuals.

We generated in silico "data" for the double exponential model $vl=A*exp(-\mu_T *t)+B*exp(-\mu_M *t)$ by randomly choosing parameters from the ranges $A \in [222000 , 54000000]$, $B \in [465 , 2000000]$, $\mu_T \in [0.6, 1.6]$, $\mu_M \in [0, 0.4]$; calculated log10(vl) at 9 time points that were chosen to be representative of the sampling scheme used by Klatt et al. (day 0, 1, 2, 3, 7, 10, 14, 21, 28) and added noise. Noise was chosen to be normally or uniformly distributed with a range of standard deviations. We then fit the model to the "data", estimated the parameters and the 95% CI on the rate of clearance of the rapidly turning over subpopulation (parameter μ_T) using the 4 methods; each bootstrap run was repeated 300 times. We repeated this 100 times and calculated what proportion of times the true value of μ_T lay within the estimated value +/- the 95%CI.

The results are shown in Figure S4. If the proportion lies above 95% then the confidence intervals are too broad, if it lies below 95% then they are too narrow.

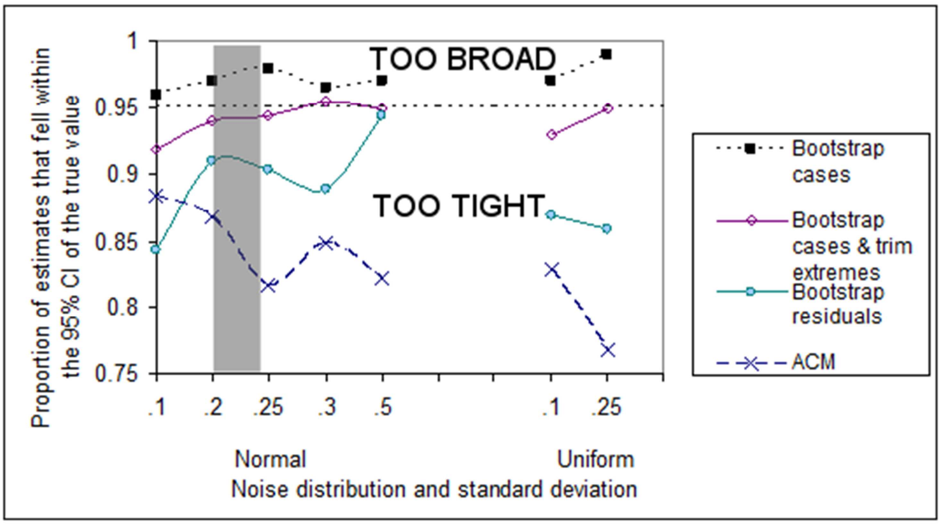

Figure S4. Evaluation of the confidence intervals produced by 4 different methods

The proportion of times the true value of μ_T lay within the estimated value +/- the 95%CI is plotted for the different noise distributions (all with mean zero, standard deviation as given on the x axis). The optimal method is the one yielding answers closest to 95%. The residuals of the model fit to the experimental data were normally distributed with mean 0.0008 and standard deviation 0.24, this is therefore the noise regime in which we are likely to be operating and where the methods need to perform best (represented by the shaded bar). For all noise distributions considered, the optimal method was to bootstrap the cases and trim the extremes. ACM and bootstrapping the residuals consistently underestimated the size of the error, bootstrapping the cases consistently over-estimated the size of the error.

Would we expect to see a trend?

Even if the measurements of the death rates of productively infected cells in control and depleted animals are not accurate enough to detect a significant difference between the two groups intuitively we might expect to at least see a trend towards higher death rates in control animals. To investigate the validity of this intuition we created in silico death rate estimates. In silico estimates for CD8-depleted animals were created by sampling 5 death rate estimates from the bootstrap estimates for the 5 CD8-depleted animals in group B as described before. In silico estimates for control animals were created by sampling 5 death rate estimates from the bootstrap runs for the SAME 5 CD8-depleted animals in group B and then adding our estimate of CTL killing in these 5 animals derived from the rise in viral load following CD8-depletion (Table S2).That is, we manually force the death rate in control animals to be higher than in depleted animals, the difference being the estimated CTL-mediated death rate. 1000 data sets were generated to represent 1000 “experiments”. Looking at all the data sets it is clear that the death rate is higher in intact animals than in depleted animals (Figure S5A). However, this cannot be discerned just by looking at individual “experiments” (see Figure S5B for the first 21 “experiments”). Indeed in only 12% of “experiments” was a trend of $p<0.2$ seen. So, we conclude that our intuition is wrong and that with the experimental design used in this study there is no reason to expect even a trend towards higher death rates in control animals.

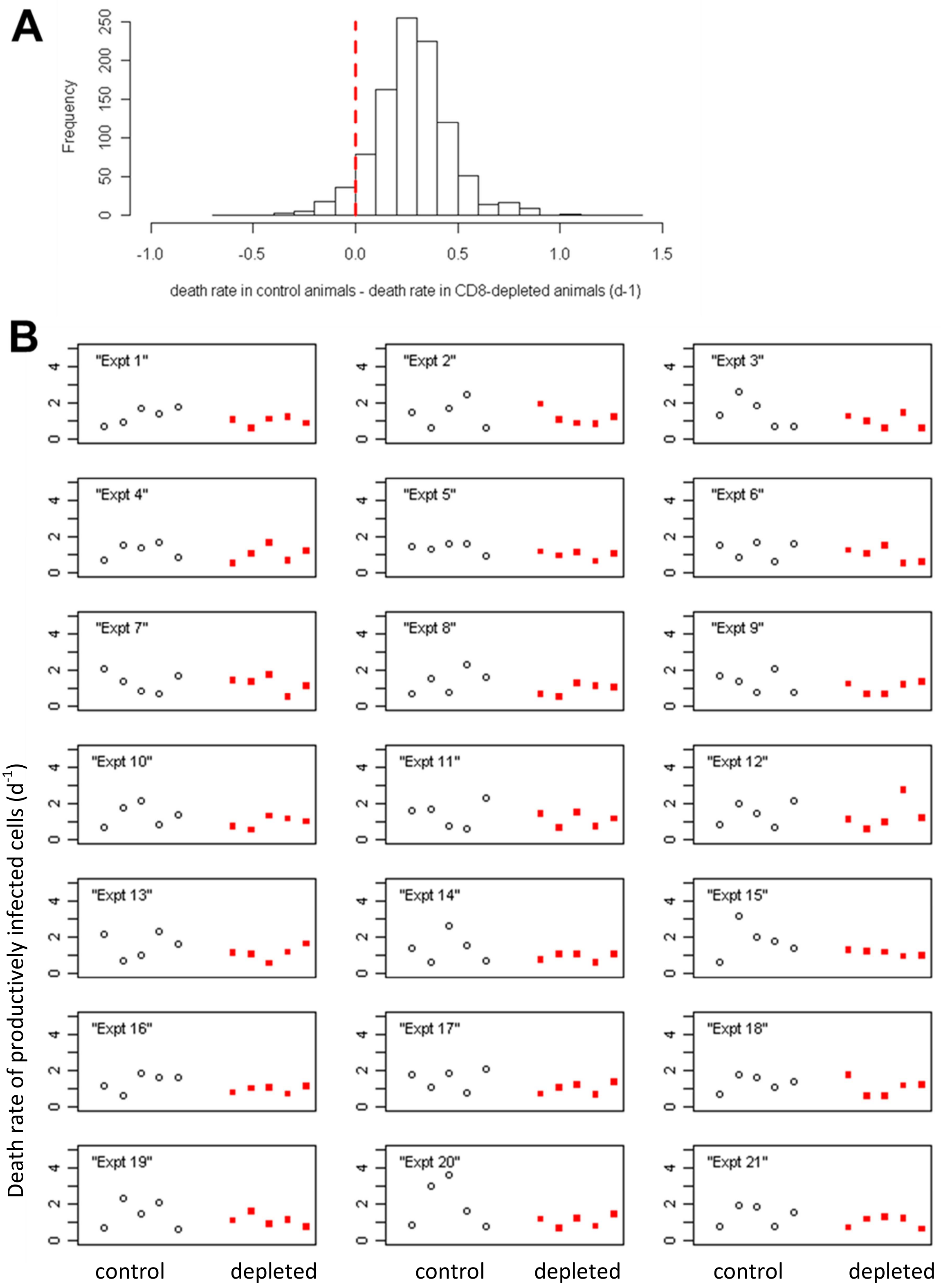

Figure S5. Death rates in 'in silico' experiments. A. Distribution of the difference in death rates between control and CD8-depleted animals. Looking at all 1000 "experiments" it is clear that the geometric mean of the death rates of productively infected cells in control animals is higher than in CD8-depleted animals i.e. the frequency distribution is markedly right shifted with a mean of 0.28d^{-1}. **B**. Death rates in the first 21 "experiments" Despite manually imposing higher death rates in control animals the difference is not apparent, even as a trend, at the level of individual "experiments".

Calculation by Wong et al

Wong et al[3] state that "the observed one log increase of VL following CD8+ T-cell depletion would require, to first approximation in the time scale studied, a 10-fold increase in productive cell lifespan for the mathematical model used here"; where the mathematical model that they fit is exponential growth from day 84 to day 91 (see Table S1 and Figure S5A,B in Wong et al [3]). They estimate that the geometric mean of the productively infected cell death rates in depleted animals is $0.56d^{-1}$ and that the difference in death rates of infected cells between control and depleted macaques is at most $0.496d^{-1}$ i.e the small difference between decline of viral load post ART in depleted and control macaques supports at most a two-fold increase in lifespan. They therefore conclude that the large increase in infected cell lifespan implied by the rate of increase of viral load following CD8 depletion is incompatible with the small difference in infected cell lifespan post ART.

However, the calculation of the increase in viral load and the increase in lifespan are in different time units: the 10-fold increase in viral load is not observed in one day but over several days. It is necessary to compare the per day increase in viral load with the per day increase in lifespan. Wong et al report a rate of increase in viral load in fully depleted macaques (Table 1 in Wong et al [3]) of $0.39d^{-1}$ (in the model they use, this is equivalent to a 10-fold increase in viral load in 5.9 days); this increase of $0.39d^{-1}$ is well within their estimate of $0.496d^{-1}$. This indicates that the change in viral load following depletion is less that the maximum difference in death rates supported by their data.

Group			Lytic models				Non-lytic models			
		**Animal**	**i**	**ii**	**iii**	**iv**	**i**	**ii**	**iii**	**iv**
	**A**	**RAj7**	0.00	6.07	12.64	51.68	2.58	7.77	6.34	11.99
		**RLi6**	9.33	11.03	44.99	31.27	13.14	0.00	14.92	3.94
		**RRf6**	34.15	45.42	46.95	37.28	18.40	0.00	23.05	6.79
	**B**	**RSq8**	9.35	7.83	46.17	16.99	0.00	4.54	6.95	6.39
		**RUe7**	24.47	32.95	59.64	80.98	7.77	0.00	14.58	21.81
		**XHB**	14.22	18.36	45.48	27.31	6.74	0.00	7.38	11.85
		**RWf7**	26.52	73.04	38.51	37.27	2.47	0.00	25.93	21.88
	**†A**	**RPp6†**	0.00	16.76	33.65	49.79	0.02	16.83	0.03	16.67
	**†B**	**RZl5†**	0.00	1.57	14.65	15.43	0.48	1.38	0.22	1.20
		**RMm6†**	9.78	80.02	43.00	68.49	0.00	62.74	9.53	79.02

Table S3 Comparison of lytic and non-lyic model fits.

The 4 lytic models are i) a basic model of lytic control ii) an extension of the basic model to include two populations of productively infected cells iii) a model following Klenerman et al in which SIV is cytopathic and iv) a model following Althaus et al in which CD8+ T cell killing is limited to the early non-productive stage of the viral lifecycle. The 4 non-lytic control models were: i) a model in which non-lytic factors reduced new infection events e.g. by production of beta-chemokines ii) an extension of model i to include two populations of productively infected cells iii) a model in which non-lytic factors reduce virion production iv) an extension of model iii to include two populations of productively infected cells.

Table S3 shows the AIC$_c$ of the model minus the AICs of the best fitting model for that animal. A large difference represents a poor fit. As a rule of thumb a difference of < 2 suggests substantial evidence for both models, values between 3 and 7 indicate that the model with the worse fit has considerably less support, whereas a difference > 10 indicates that the model with the worse fit is very unlikely [4]. The best fit model is shaded, comparable models are shown in bold.

The best-fitting non-lytic model (non-lytic model ii) was compared with each of the lytic models in turn. In every case the non-lytic model provided a significantly better fit (lytic model i higher AICc 6/7 cases P=0.043, mean difference in AICc=15; lytic model ii higher AICc 6/7 cases P=0.028, mean difference in AICc=26; lytic model iii higher AICc 7/7 cases P=0.018, mean difference in AICc=40; lytic model iv higher AICc 7/7 cases P=0.018, mean difference in AICc=39. All P values 2 tailed paired Mann-Whitney). The last three animals in the list were euthanized after early chronic infection, receiving either ART-treatment alone or the combination of CD8+ T cell depletion and ART. These animals were excluded from the analysis as, in each case, they had received only half the treatment (i.e. just ART or just ART/depletion) so there was insufficient data to constrain the fits. Including these 3 animals in the analysis did not change the result (P=0.028, two tailed paired Mann-Whitney as before).

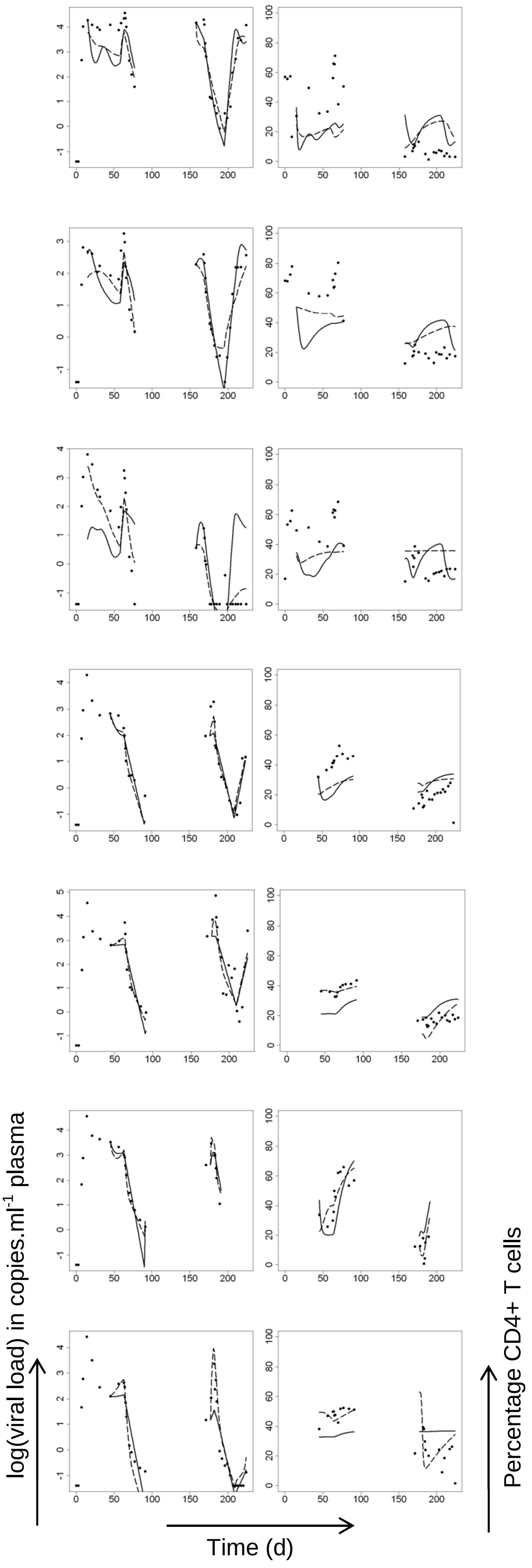

Figure S6. Experimental data and model fits. Experimental viral load and percentage CD4+ T cells (filled dots on left column and right column respectively) predicted by the best fitting non-lytic model (non-lytic model ii; solid lines) and the best-fitting lytic model (lytic model i; dashed lines) for all data sets. These models have a different number of parameters (10 and 8 respectively), favouring the model with the larger number of parameters in terms of sum of squared residuals. Model selection was based in the AIC_c, which provides a more objective comparison. Please note that for animals in Group B data for CD4+ and CD8+ T cells was only available from 45 days after infection, hence viral load data before this time point have not been fitted

Reference List

1. Dixit NM, Markowitz M, Ho DD, Perelson AS (2004) Estimates of intracellular delay and average drug efficacy from viral load data of HIV-infected individuals under antiretroviral therapy. Antiviral Therapy 9: 237-246.

2. Markowitz M, Louie M, Hurley A, Sun E, Di Mascio M (2003) A novel antiviral intervention results in more accurate assessment of human immunodeficiency virus type 1 replication dynamics and T-Cell decay in vivo. J Virol 77: 5037-5038.

3. Wong JK, Strain MC, Porrata R, Reay E, Sankaran-Walters S et al. (2010) In Vivo CD8+ T-Cell Suppression of SIV Viremia Is Not Mediated by CTL Clearance of Productively Infected Cells. PLoS Pathog 6.

4. Burnham, K. P. and Anderson, D. R. (2002) Model Selection and Multimodel Inference: a practical information-theoretic approach. New York: Springer. 488 p.
